# Metformin combined with sodium dichloroacetate promotes B leukemic cell death by suppressing anti-apoptotic protein Mcl-1

**DOI:** 10.18632/oncotarget.7879

**Published:** 2016-03-03

**Authors:** Rebecca Voltan, Erika Rimondi, Elisabetta Melloni, Paola Gilli, Valerio Bertolasi, Fabio Casciano, Gian Matteo Rigolin, Giorgio Zauli, Paola Secchiero

**Affiliations:** ^1^ Department of Morphology, Surgery, Experimental Medicine and LTTA Centre, University of Ferrara, Ferrara, Italy; ^2^ Department of Life Sciences, University of Trieste, Trieste, Italy; ^3^ Department of Chemical and Pharmaceutical Sciences, University of Ferrara, Ferrara, Italy; ^4^ Department of Medical Sciences, University of Ferrara-Arcispedale S. Anna, Ferrara, Italy

**Keywords:** metformin, sodium dichloroacetate, B chronic leukemic cells, Mcl-1, apoptosis

## Abstract

Metformin and the mitochondrial targeting dichloroacetate (DCA) have recently received attention due to their ability to inhibit anaerobic glycolysis, which renders most cancer cells resistant to apoptosis induction. We observed that Metformin alone exhibited a dose-dependent anti-leukemic activity in both B leukemic cell lines and primary B-chronic lymphocytic leukemia (B-CLL) patients' cells and its anti-leukemic activity was enhanced when used in combination with DCA. In order to overcome the problems of poor bioavailability and cellular uptake, which limit DCA efficacy, we have designed and synthetized cocrystals consisting of Metformin and DCA (Met-DCA) at different stoichiometric ratios. Of note, the MetH_2_^++^•2DCA^−^ cocrystal exhibited enhanced *in vitro* anti-leukemic activity, with respect to the treatment with the mix consisting of Metformin plus DCA. In particular, the treatment with the cocrystal MetH_2_^++^•2DCA^−^ induced a synergistic apoptotic cell death coupled to a marked down-modulation of the anti-apoptotic Mcl-1 protein. Taken together, our data emphasize that innovative compounds based on Metformin-DCA combination merit to be further evaluated as chemotherapeutic agents for the treatment of B-CLL.

## INTRODUCTION

Most cancers are characterized by enhanced glycolytic flux for ATP production, enhanced glucose to lactate conversion and reduced mitochondrial oxidative phosphorylation, even under aerobic conditions [1-3]. Interestingly, this cancer-specific metabolic remodeling can be reversed by dichloroacetate (DCA), a mitochondria-targeting small molecule able to penetrate most tissues after oral administration [4]. DCA is a generic drug with low price, which has been used for human treatments for more than 30 years and has received renovated attention because of interesting preclinical antitumoral characteristics, assessed in solid tumor cell lines [4-7], and relatively low toxicity on normal cells. Some recent studies of our and other groups have demonstrated that DCA is also effective against hematological malignancies, such as multiple myeloma [8] and in particular, for the purpose of this study, against B-chronic lymphocytic leukemia (B-CLL) cells [9, 10].

Among the great variety of anti-cancer drugs, another inexpensive and safe drug, which has recently revealed a potential anti-leukemic activity, is Metformin [11-14]. This molecule represents the most commonly prescribed drug for type 2 diabetes mellitus [11]. Anyhow, in recent years, multiple lines of evidence have provided support for the hypothesis that treatment with Metformin results in decreased incidence, progression, and mortality of different human cancers. Moreover, a number of *in vitro* studies have documented the antiproliferative, anti-invasive, and antimetastatic effects of Metformin in multiple cancer cell types [15-18]. Interestingly, DCA and Metformin share several mechanisms, potentially involved in their anticancer activity, by disrupting mitochondrial respiratory chain complex and decreasing the ATP synthesis [19].

On these bases, the aim of the present study was to evaluate the potential therapeutic perspectives of Metformin plus DCA as innovative anti-leukemic drug combination. Herein, we have evaluated the *in vitro* effects of Metformin used alone and in combination with DCA on B-leukemic cells, including primary B-CLL patient cells, by assessing cell viability, cell cycle progression, apoptosis, as well as the expression of apoptotic signaling modulators. Of note, to improve the efficacy of the drug combination, we have designed, synthetized and functionally validated Metformin-DCA cocrystals.

## RESULTS

### Metformin promotes cytotoxicity in B leukemic cell lines and in primary B-CLL cells

**Figure 1:**
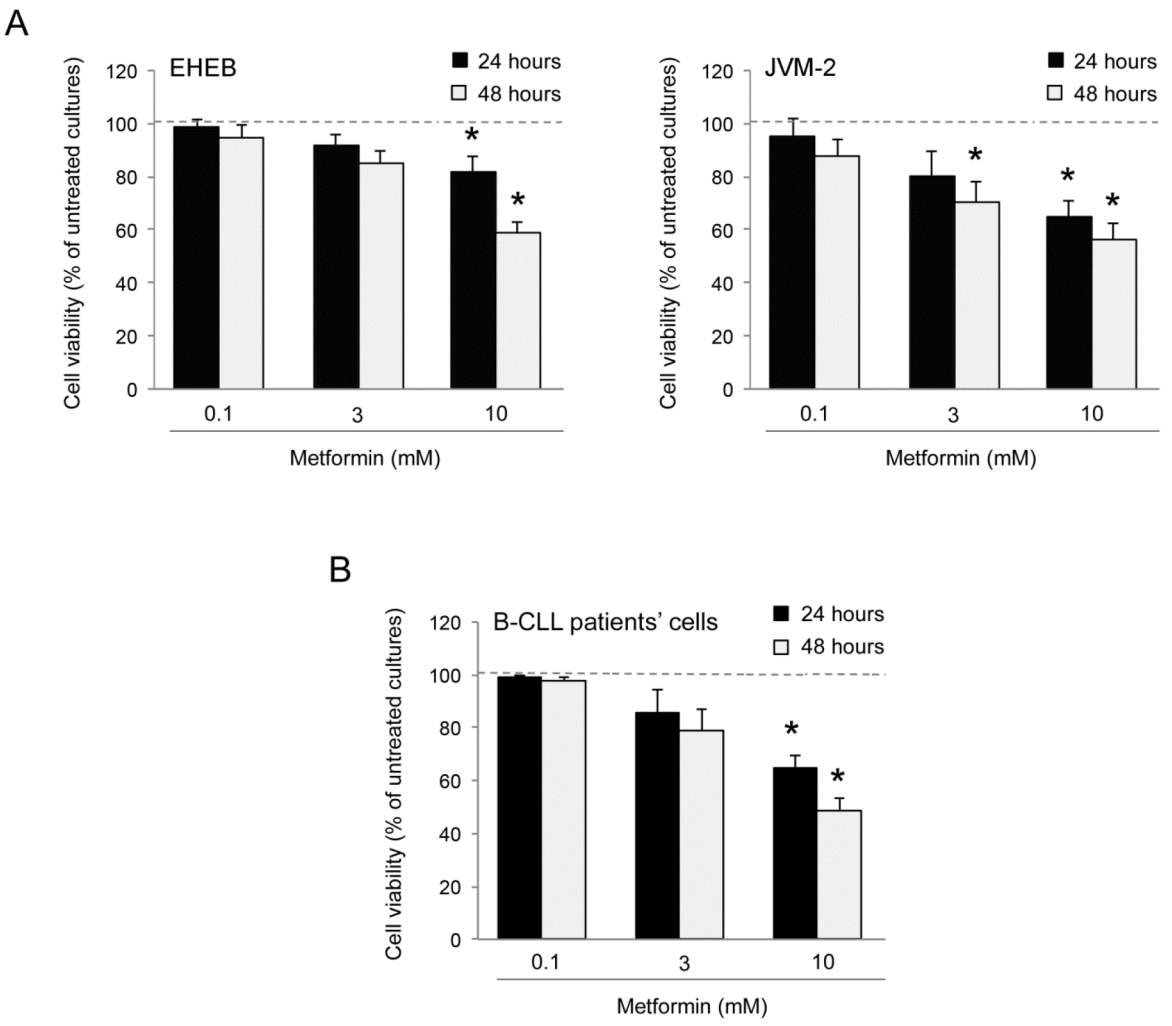
Cytotoxicity induced by Metformin in B leukemic cells The B leukemic cell lines EHEB and JVM-2 **A.** as well as B-CLL patients' leukemic cells (n=6) **B.** were exposed to serial doses of Metformin (range 0.1-10 mM) before analysis of cell toxicity. In A and B, cell viability in response to Metformin was calculated at both 24 and 48 hours of treatment as percentage with respect to the control vehicle cultures (set to 100% for each cell line). Data are reported as mean values ±SD of results of at least six independent experiments. The asterisk indicates p<0.05 with respect to the untreated cultures.

In the first set of experiments, we have evaluated the *in vitro* effect of Metformin on B leukemic cell lines (EHEB and JVM-2), as well as on primary B-CLL patient cell samples. All leukemic cells were characterized by having a p53^wild-type^ status, a feature typical of the majority of the B-CLL at diagnosis [20-25]. Treatment with Metformin exhibited a dose- and time-dependent cytotoxicity on both B-leukemic cell lines (Figure 1A) as well as on B-CLL patient cell cultures (Figure 1B). Of note, the IC_50_ mean values (±SD) calculated after 48 hours of treatment in B leukemic cell lines (11.58±0.77 mM) and B-CLL patient derived cell cultures (10.17±1.04 mM) were comparable.

### Anti-leukemic activity of Metformin plus DCA and of Met-DCA cocrystals

**Figure 2:**
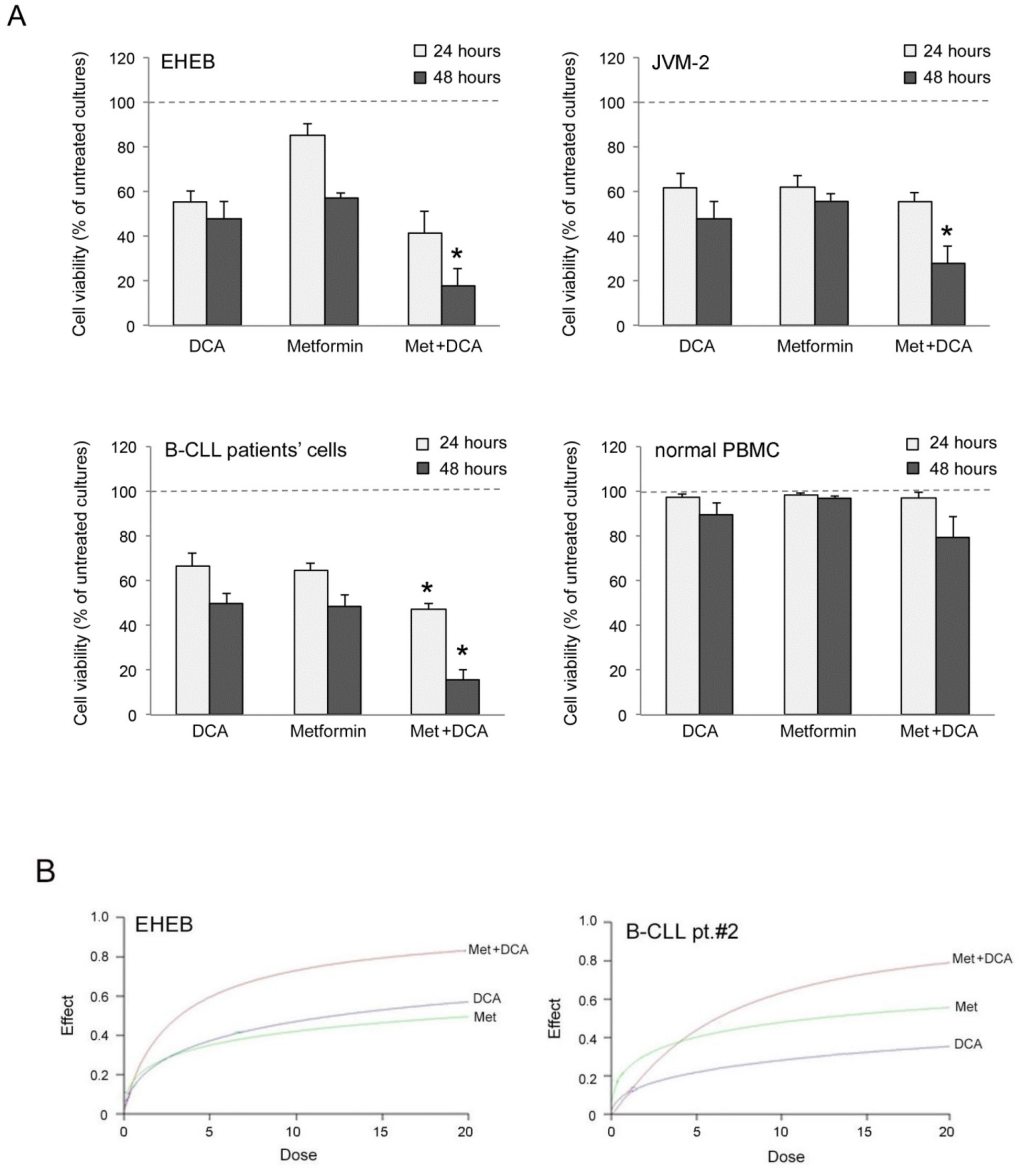
Evaluation of cell cytotoxicity in response to Metformin in combination with DCA in B-CLL leukemic cells The B leukemic cell lines EHEB and JVM-2, B-CLL patients' leukemic cells (n=5) and normal PBMC from healthy donors (n=3) were exposed to DCA (range of 0.2 to 20 mM) or Metformin (range of 0.1 to 10 mM) used either alone or in combination for 24 and 48 hours. In **A.** cell viability in response to Metformin (10 mM) and DCA (20 mM), used alone or in combination, was calculated as percentage with respect to the control vehicle cultures (set to 100% for each cell line). Data are reported as the mean±SD of results of at least three independent experiments; the asterisk indicates a significant difference (p<0.05) with respect to the treatment with the single agents. In **B.** dose-effect plots of drug efficacy, documenting a synergistic effect of the combination Metformin plus DCA, are shown for representative experiments performed on EHEB cells and on one B-CLL patient sample. The decrease of cell viability, labeled “effect” on the Y-axis, was determined in assays done at least twice and in duplicate.

Starting from our recent studies documenting anti leukemic activity of DCA towards B-CLL [9, 10], in the next group of experiments we have explored the potentiality of using Metformin in combination with DCA. B-CLL cells were treated with Metformin and DCA (used in the range of 0.1-20 mM) as single agents and in combination. In particular, leukemic cells were treated with serial concentrations of Metformin and DCA at a constant Metformin:DCA ratio (either 1:1 or 1:2) for data analysis by the method of Chou and Talalay [26]. Combined treatment with Metformin plus DCA, at 1:2 ratio, resulted in significantly (p<0.05) enhanced cytotoxicity with respect to the single agents in both B leukemic cell lines as well as in primary B-CLL patient samples (Figure 2A), with a synergistic effect (Figure 2B) documented by an average Combination Index (CI) value <1. On the other hand, no significant cytotoxicity was observed in normal peripheral blood cells exposed to the single drugs, confirming literature data [9, 27], as well as to the Metformin plus DCA combination (Figure 2A). Starting from these results and considering that DCA molecule exhibits poor bioavailability and cellular uptake, we have synthetized new molecules consisting of cocrystals of Metformin and DCA in different stoichiometric ratios: MetH^+^•DCA^−^ (1:1; Figure 3A) and MetH_2_^++^•2DCA^−^ (1:2; Figure 3B). When tested on leukemic cell lines and B-CLL patient derived primary cells, these compounds exhibited *in vitro* anti-leukemic activity. In particular, maximal cytotoxic effects were observed when cell cultures were treated with the cocrystal MetH_2_^++^•2DCA^−^ (from now on, named Met-DCA cocrystal), which exhibited an enhanced (p<0.05) activity with respect to the treatment with a mix of the two reference drugs, used at the appropriate concentrations (Figure 4). Analysis of the cell cycle profile (Figure 5A) revealed that the cytotoxicity induced by the treatment with the Met-DCA cocrystal was effective in increasing the cytostatic effect induced by DCA (id, accumulation in G1 phase and reduction in S phase of the cell cycle). Moreover, the Met-DCA cocrystal was particularly effective as pro-apoptotic molecule resulting in a significant (p<0.05) increase of the degree of apoptosis with respect to the treatment with Metformin and DCA used as single agents (Figure 5B-5C). Of note, the pro-apoptotic effects of the Met-DCA cocrystal were significantly (p<0.05) higher also with respect to the treatment with the mix of Metformin plus DCA (Figure 5B-5C).

**Figure 3:**
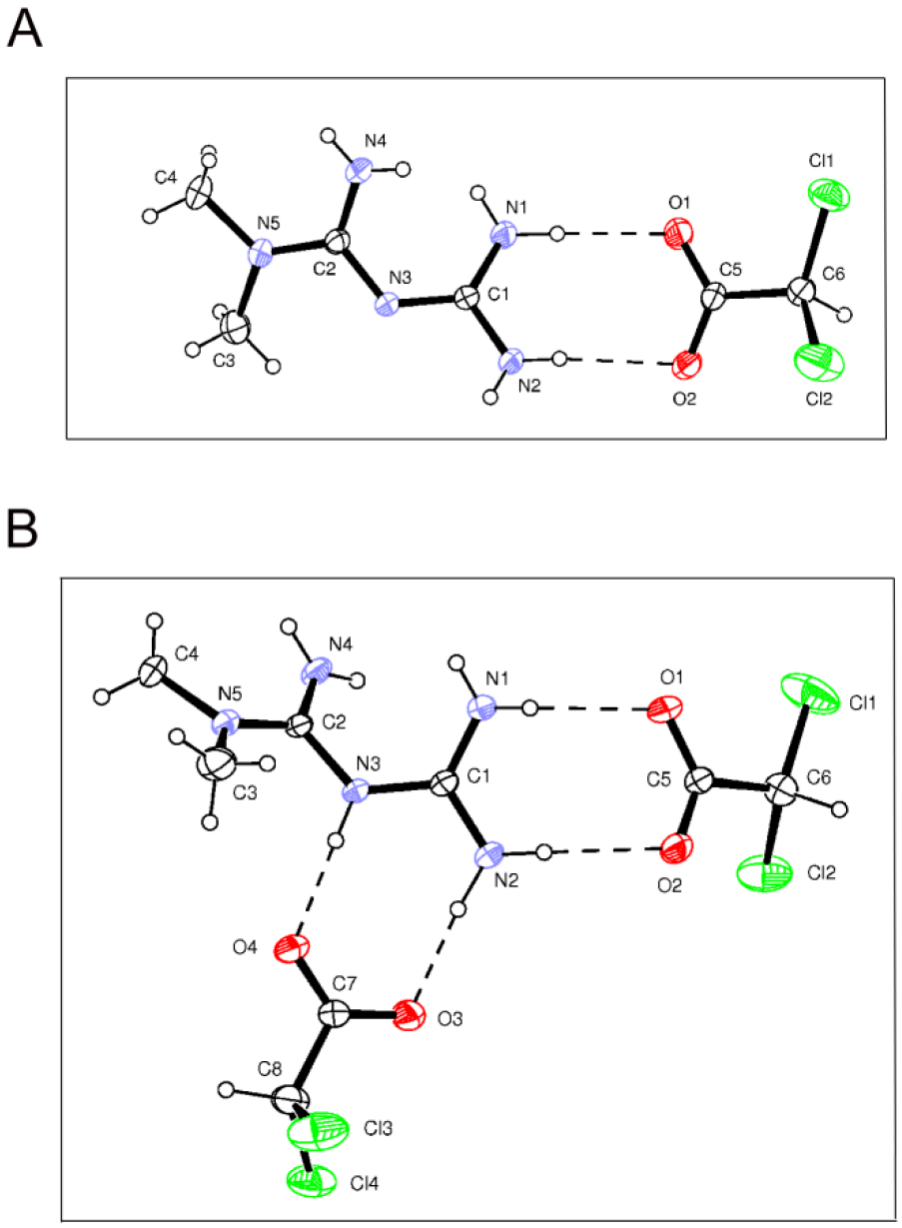
Structure of Metformin and DCA cocrystals Drawings of Met-DCA 1:1 (MetH^+^•DCA^−^) **A.** and Met-DCA 1:2 (MetH_2_^++^•2DCA^−^) **B.** cocrystals are shown (ORTEP view with the thermal ellipsoids at 30% probability).

**Figure 4:**
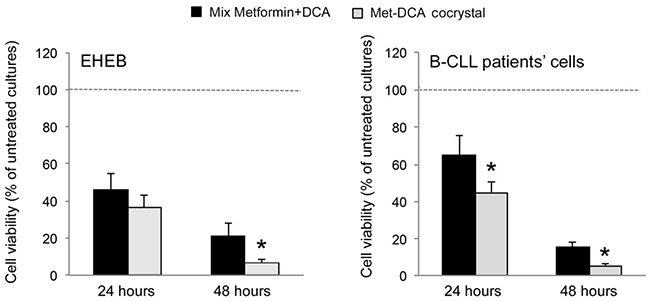
Comparative evaluation of the anti-leukemic activity of Metformin plus DCA (mix) and of the Met-DCA cocrystal MetH_2_^++^•2DCA^−^ The B leukemic cell lines EHEB and JVM-2 as well as B-CLL patients' cells (n=5) were exposed to a mix of Metformin (10 mM) plus DCA (20 mM) or to the Met-DCA cocrystal MetH_2_^++^•2DCA^−^ (10 mM) before analysis of cell viability at 24 and 48 hours. Cell viability was calculated as percentage with respect to the control vehicle cultures (set to 100%). Data are reported as the mean±SD of results from at least four independent experiments. The asterisk indicates a significant difference (p<0.05) with respect to the treatment with the mix. Considering that results were comparable in both cell lines, only EHEB results are shown as representative.

**Figure 5:**
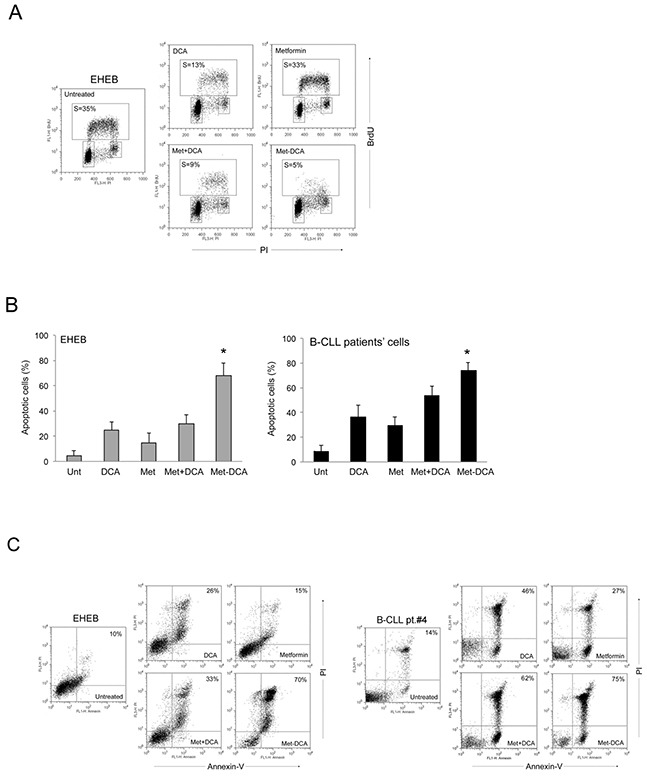
Mechanism of the anti-leukemic activity of the Met-DCA cocrystal MetH_2_^++^•2DCA^−^ on B leukemic cells The B leukemic cell lines EHEB and JVM-2 as well as B-CLL patients' cells were exposed to Metformin (10 mM) and DCA (20 mM), used either alone or in combination, and to the Met-DCA cocrystal MetH_2_^++^•2DCA^−^ (10 mM) before cytofluorimetric analysis of cell cycle progression **A.** and apoptosis induction **B-C.** In A, representative cell-cycle profiles analyzed by BrdU incorporation after 24 hours of the indicated treatments. The rectangles represent the cells in the different (G0/G1, S, G2/M) phases of the cell cycle and the percentage of cells in S-phase is indicated for each treatment. Plots representative of three independent experiments yielding equivalent results are shown. In B, the percentages of apoptosis after 48 hours of treatment are reported as the mean±SD of results from three independent experiments. The asterisk indicates a significant difference (p<0.05) with respect to the treatment with the mix Metformin plus DCA. In C, representative plots of apoptotic cells analyzed by flow-cytometry after annexin-V/PI staining. The percentage of apoptotic cells is indicated for each treatment. Considering that results were comparable in both cell lines, only EHEB results are shown as representative.

### The Met-DCA cocrystal-induced cell death is accompanied by Mcl-1 down-regulation

**Figure 6:**
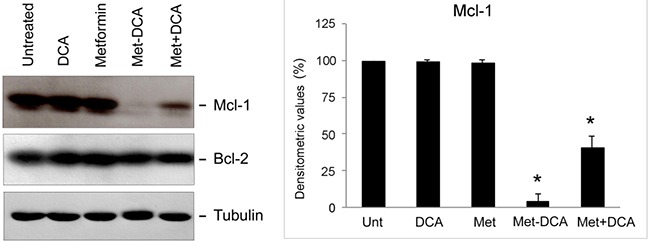
Mcl-1 down-regulation in p53 wild-type leukemic cells upon treatment with Metformin plus DCA combination and with the Met-DCA cocrystal MetH_2_^++^•2DCA^−^ Equal amounts of cell lysates, obtained from JVM-2 cell lines treated for 24 hours as indicated, were analyzed for Mcl-1 and Bcl-2 protein levels by Western blotting. Tubulin staining is shown as a loading control. Blottings representative of at least three independent experiments yielding equivalent results in both JVM-2 and EHEB cells, are shown. Mcl-1 bands' density was quantified using Image Quant TL software and normalized to the untreated band (set at 100%) on the same membrane. Data are reported as mean±SD, The asterisk indicates a significant difference (p<0.05) with respect to the treatment with the single treatments.

**Figure 7:**
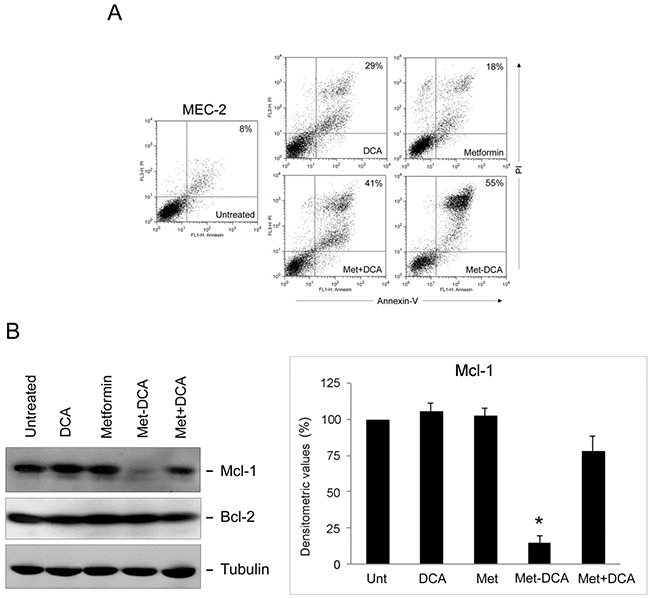
Cell cytotoxicity and Mcl-1 down-modulation on p53 mutated B leukemic cells in response to treatment with Metformin plus DCA combination and with the Met-DCA cocrystal MetH_2_^++^•2DCA^−^ MEC-2 p53^mutated^ leukemic cells were exposed to Metformin (10 mM), DCA (20 mM), alone or in combination, or to the Met-DCA cocrystal MetH_2_^++^•2DCA^−^(10 mM) before analysis. In **A.** representative plots of apoptotic cells analyzed by flow-cytometry after Annexin-V/PI staining after 48 hours of treatment are shown. The percentage of apoptotic cells is indicated for each treatment. Results representative of at least three independent experiments yielding equivalent results are shown. In **B.** equal amounts of cell lysates, obtained from MEC-2 cell lines treated for 24 hours as indicated, were analyzed for Mcl-1 and Bcl-2 protein levels by Western blotting. Tubulin staining is shown as a loading control. Blottings representative of at least three independent experiments yielding equivalent results are shown. Bands' density was quantified using Image Quant TL software and normalized to the untreated band (set at 100%) on the same membrane. Data are reported as mean±SD, The asterisk indicates a significant difference (p<0.05) with respect to the treatment with the single treatments.

In order to evaluate the molecular basis explaining the antitumoral effects of the Met-DCA cocrystal, after *in vitro* treatments, we have analyzed the expression profile of intracellular effectors known to stimulate/suppress leukemic cell survival. In particular, considering previous studies on the molecular effects of Metformin, alone or in combination with other therapeutic compounds, in solid tumor cell models and in multiple myeloma cells [28-30], we have first investigated the expression levels of Mcl-1. Of note, Mcl-1 is one of the most important anti-apoptotic Bcl-2 family member, which induces CLL cell survival and therefore is associated with CLL response to the treatments and disease progression [31, 32]. As shown in Figure 6, in our experimental models, Mcl-1 protein levels were significantly decreased upon 24 hours of treatment with the combination of Metformin plus DCA. Of interest, maximal down-regulation was observed by treatment with the Met-DCA cocrystal (Figure 6). It has to be underlined that the down-modulation of Mcl-1, coupled to the induction of apoptosis, in response to Met-DCA cocrystal was documented not only in B leukemic cells with a p53^wild-type^ status, but also in the B lymphoblastoid cell line MEC-2 harboring mutated p53 (Figure 7). Conversely, the levels of the pro-apoptotic Bcl-2 protein did not change upon drug treatments neither in p53^wild-type^ (Figure 6) nor in p53^mutated^ cells (Figure 7).

**Figure 8:**
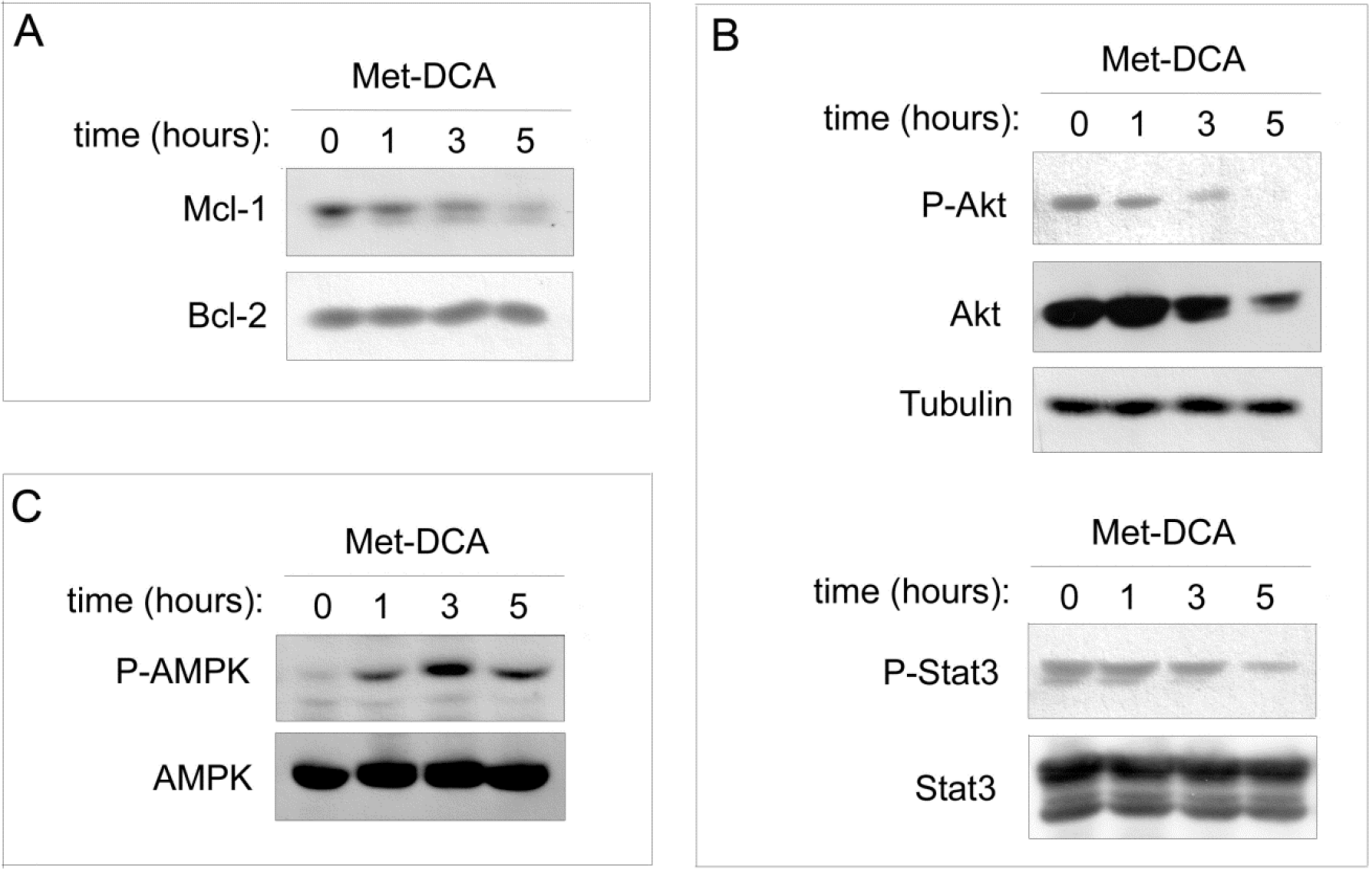
Effects of the Met-DCA cocrystal MetH_2_^++^•2DCA^−^ on different molecular signaling pathways JVM-2 cells were treated with the Met-DCA cocrystal MetH_2_^++^•2DCA^−^(10 mM) for different time-points as indicated. Cell lysates were harvested and analyzed in immunoblotting using specific antibodies to detect: Mcl-1, Bcl-2 **A.** Akt, phospho-Akt (Ser473), STAT3, phospho-STAT3 (Ser727) **B.** AMPKα, phospo-AMPKα (Thr172) **C.** Tubulin staining is shown as a loading control. Representative examples of Western blotting results of three independent experiments are shown.

In the next experiments, we have analyzed Mcl-1 protein levels at early time points upon drug exposure. As shown in Figure 8A, Mcl-1 down-modulation was evident already after 3-5 hours of treatment with the Met-DCA cocrystal, well before the onset of apoptosis. In parallel, since in different cancer cells Mcl-1 is induced by Akt and STAT-3 intracellular pathways [33-35], which are implicated in B-CLL pathogenesis [12, 36-39], we have investigated the potential involvement of Akt and STAT-3 in the anti-leukemic activity of Met-DCA cocrystal. As shown in Figure 8B, down-modulation of Mcl-1 in response to the Met-DCA cocrystal was coupled to a significant down-regulation of phospho-Akt, total Akt, and phospho-STAT3 protein levels. On the other hand, in line with previous studies on Metformin [13, 14, 18], our present study documented the ability of Met-DCA cocrystal of stimulating the AMP-activated protein kinase (AMPK) pathway (id, AMPK phosphorylation; Figure 8C), an energy-sensing system associated with Mcl-1 down-modulation in different cell models [40-42].

## DISCUSSION

Based on FDA definition, cocrystals are solids composed of two or more molecules in the same crystal lattice held together in stoichiometric amounts by freely reversible, noncovalent forces of interaction [43]. When one of the molecules is an Active Pharmaceutical Ingredient (API) they are called pharmaceutical cocrystals and have received recent attention because of better pharmaceutical profiles over the native drugs [44]. Moreover, the concept of modifying the properties of a single API by cocrystallization with a coformer, that may be another API that improves the delivery and complements the efficacy of the reference drug, has receiving increasing interest for drug development [45].

In virtue of our previous observations documenting the cytotoxic properties of DCA towards B-CLL [9, 10], in the present *in vitro* study, we have analyzed the potential antileukemic activity of Metformin used in combination with DCA, either as a mix of the two compounds or as a cocrystal composed of Metformin and DCA (1:2). We have documented the ability of the Metformin and DCA combination and, even more, of the Met-DCA cocrystal, in promoting cytotoxicity in both B leukemic cell lines and B-CLL patient derived cell cultures as result of both a cytostatic and pro-apoptotic effect.

Beyond the plausible effects on several physicochemical properties (such as solubility), we observed that the use of the Met-DCA cocrystal enhanced the biological effect of the mix composed by the two drugs, probably as a result of an improved cellular uptake of the cocrystal compared to the reference drugs. We could argue that the molecule Metformin may act as a carrier for DCA, which indeed requires high dosage in therapies because of the low bioavailability [46]. The concept of an enhanced bioavailability of the cocrystal, associated to the advantage of administration of a single pharmaceutic compound instead of the separated reference drugs, certainly offers better opportunities for future *in vivo* preclinical assessments (i.e., investigations in animal models) and for the translation of the Met-DCA, compound based on two inexpensive and safe drugs, to clinical applications.

In the effort to elucidate the molecular mechanism of action of Met-DCA cocrystal, we investigated whether the anti-apoptotic Bcl-2 family members might represent targets for Met-DCA cocrystal. Our data documented a correlation between Mcl-1 down-regulation and cytotoxicity induced by the Metformin plus DCA combination and Met-DCA cocrystal in B leukemic cells, with an early down-regulation of Mcl-1 (but not of Bcl-2), associated to a concomitant down-regulation of phospho-Akt, total Akt, and phospho-STAT3 protein levels, which anticipated the onset of apoptosis. These data are particularly interesting since alteration of the intrinsic pathway of apoptosis is a major target of cancer cells to evade apoptosis, and often this goal is achieved through the increase in the expression level of Bcl-2 and/or Mcl-1 [47]. In this respect, several Bcl-2 inhibitors (such as navitoclax and venetoclax) have shown efficacy as chemotherapy agents in clinical trials, by binding with high affinity Bcl-2 and Bcl-Xl [48, 49]. However, none of these compounds can bind and antagonize the prosurvival activity of the Mcl-1 Bcl-2 family member, which promote the development of many cancers and/or in their resistance to chemotherapy [50]. It is also a key factor in the resistance of malignant cells to navitoclax and venetoclax. Interestingly, it has been recently shown that the Notch-dependent up-regulation of Mcl-1 promotes survival of B-CLL [51] and thus it represents an axis for therapeutic interventions in the perspective to eradicate the leukemic clone.

Since Mcl-1 silencing has been found to elicit tumor regression and cell death in various cancer models [52], including B-CLL [53], our data provide the rationale for further evaluating the use of Met-DCA cocrystal (also in combination with the Bcl-2 specific targeting drug venetoclax) for the treatment of B-CLL, also in virtue of the potential anti-leukemic activity of Met-DCA independently of the p53 status.

## MATERIALS AND METHODS

### Cell cultures and treatments

The B leukemic cell lines EHEB, JVM-2 and MEC-2 were purchased from DSMZ (Deutsche Sammlung von Mikroorganismen und Zellkulturen GmbH, Braunschweig, Germany). EHEB and JVM-2 cell lines were routinely cultured in RPMI-1640, whereas MEC-2 cells were maintained in IMDM, all supplemented with 10% FBS, L-glutamine and Penicillin/streptomycin (all from Gibco, Grand Island, NY). For experiments with primary cells, peripheral blood samples were collected in heparin-coated tubes from either normal blood donors or from B-CLL patients following informed consent, in accordance with the Declaration of Helsinki and in agreement with institutional guidelines (University-Hospital of Ferrara). The diagnosis of B-CLL was made by peripheral blood morphology and immunophenotyping and all patients had been without prior therapy at least for three months before blood collection. Peripheral blood mononuclear cells (PBMC) were isolated by gradient centrifugation with lymphocyte cell separation medium (Cedarlane Laboratories, Hornby, ON). T lymphocytes, NK lymphocytes, granulocytes and monocytes were negatively depleted from peripheral blood leucocytes (PBL) with immunomagnetic microbeads (MACS microbeads, Miltenyi Biotech, Auburn, CA), with a purity >95% of resulting CD19^+^ B-CLL population, assessed by flow cytometry, as previously described [54, 55]. For *in vitro* assays, both freshly isolated or thawed B-CLL cells, previously resuspended in freezing solution (10% DMSO and 90% FBS) and cryopreserved in liquid nitrogen, were seeded in RPMI containing 10% FBS, L-glutamine and Penicillin/Streptomicin (Gibco). Freezing/thawing of B-CLL primary cells did not influence sensitivity to the treatments, since we assessed in parallel fresh and frozen/thawed cells of three patients without observing any significant cytotoxic differences.

### Culture treatments, assessment of cell viability, apoptosis and cell cycle profile

Leukemic cells were treated with serial doses of Metformin hydrochloride (1,1-Dimethylbiguanide hydrochloride, Sigma-Aldrich, St Louis, MO; range 0.1-10 mM), DCA (Sigma-Aldrich; range 0.2-20 mM), used alone or in combination, or with a Met-DCA cocrystal that was synthesized to combine Metformin plus DCA.

At different time points after treatment, cell viability was examined by Trypan blue dye exclusion and MTT (3-(4,5-dimethilthiazol-2yl)-2,5-diphenyl tetrazolium bromide) colorimetric assay (Roche Diagnostics Corporation, Indianapolis, IN) for data confirmation, as previously described [56, 57]. IC_50_ values were calculated from dose-response curves constructed by plotting cell survival (%) versus drug concentration.

Levels of apoptosis were quantified by Annexin V-FITC/propidium iodide (PI) staining (Immunotech, Marseille, France) followed by analysis using a FACSCalibur flow cytometer (Becton-Dickinson, San Jose, CA). To avoid non-specific fluorescence from dead cells, live cells were gated tightly using forward and side scatter, as described [58, 59]. The cell cycle profile was analyzed by flow cytometry after 5-bromodeoxyuridine (BrdU) incorporation, as described [60].

### Synthesis of the multicomponent Met-DCA cocrystals

All adducts of Metformin with DCA (Met-DCA 1:1, MetH^+^•DCA^−^; Met-DCA 1:2, MetH_2_^++^•2DCA^−^) were prepared using reagents and solvents purchased from a commercial source (Sigma Aldrich) and used without further purification. In particular, synthesis was carried out as follows:

- Met-DCA 1:1 (MetH^+^•DCA^−^). A methanol solution (30 mL) of metformin hydrochloride (588.7 mg, 3.55 mmol) and sodium dichloroacetate (536,4 mg, 3.55 mmol) was stirred for 12 hours at room temperature. The solvent was removed under reduced pressure and 2-propanol (100 mL) was added to the reaction solid residue. Suitable crystals for X-ray diffraction were obtained by slow evaporation at room temperature of clear solutions of neutral metformin and dichloroacetic acid dissolved in 1:1 molecular ratio in a 50:50 (v:v) methanol/n-pentanol mixture.
- Met-DCA 1:2 (MetH_2_^++^•2DCA^−^). Equimolar amounts of sodium dichloroacetate (512.8 mg, 3.4 mmol) and dichloroacetic acid (438,1 mg, 3.4 mmol) were added to a methanol solution (30 mL) of metformin hydrochloride (562.7 mg, 3.4 mmol) and stirred at room temperature. After 12 hours the solvent was evaporated under reduced pressure and 2-propanol (100 mL) was added to the solid sediment. Suitable crystals for X-ray diffraction were obtained by slow evaporation at room temperature of clear solutions of neutral metformin and dichloroacetic acid dissolved in 1:1 and 1:2 molecular ratio, respectively, in a 50:50 (v:v) methanol/n-pentanol mixture.

The identity of chemical composition and crystal phase of the compounds obtained as powder in larger amount was assessed by comparing the experimental X-ray powder diffraction (XRPD) patterns with those calculated using the program Mercury [x2] from the structures determined by single-crystal X-ray diffraction. The crystal data of compounds Met-DCA 1:1 and Met-DCA 1:2 were collected at room temperature using a Nonius Kappa CCD diffractometer with graphite monochromated Mo-Kα radiation (Bruker-Nonius, Milan, Italy). [61, 62].

### Western blotting analyses

For Western blotting analysis, cells were lysed as previously described [63, 64]. Protein determination was performed by BCA Protein Assay (Thermo Scientific, Rockford, IL). Equal amounts of protein for each sample were migrated in SDS-polyacrylamide gels and blotted onto nitrocellulose filters. The following Abs were used: anti-Mcl-1 (S-19) and anti-Bcl-2 (100) from Santa Cruz Biotechnology (Santa Cruz, CA); anti-phospho-AMPKα (Thr172, D79.5E), anti-AMPKα, anti-phospho-STAT3 (Ser727) and anti- STAT3 from Cell Signalling (Danvers, MA); anti-phospho-Akt1/PKBα (Ser473) from Merck Millipore (Darmstadt, Germany); anti AKT/PKBα from BD; anti-tubulin from Sigma-Aldrich. After incubation with anti-mouse or anti-rabbit IgG horseradish peroxidase-conjugated secondary Abs (Sigma-Aldrich), specific reactions were revealed with the ECL Lightning detection kit (Perkin Elmer, Waltham, MA). The estimation of the densitometry values of bands was obtained by the ImageQuant TL software (GE Healthcare, Buckinghamshire, UK).

### Statistical analysis and assessment of the effect of combination treatment

The results were evaluated by using analysis of variance with subsequent comparisons by Student's t-test and with the Mann-Whitney rank-sum test. Statistical significance was defined as p<0.05. In order to investigate the effect of Metformin plus DCA combination, leukemic cells were treated with serial doses of Metformin or DCA, individually or in combination using a constant ratio (Metformin:DCA). Results were analyzed with the method of Chou and Talalay [26] to determine whether combined treatment yields greater effects than expected from summation alone: a combination index (CI) of 1 indicates an additive effect, while a CI below 1 indicates synergism. For this purpose cell viability data were analyzed with the CalcuSyn software (Biosoft, Cambridge, UK) and reported either as CI values or as dose-effect curves directly drawn by the CalcuSyn software.
